# Editorial: Understanding Social Signals: How Do We Recognize the Intentions of Others?

**DOI:** 10.3389/fpsyg.2016.00281

**Published:** 2016-02-23

**Authors:** Sebastian Loth, Jan P. De Ruiter

**Affiliations:** Linguistics and Literary Studies, Psycholinguistics, Bielefeld UniversityBielefeld, Germany

**Keywords:** social signals, social communication, intention recognition, human–robot interaction, human–human interaction, experimental methods, interaction design

Humans interact with each other seamlessly, smoothly, and without obvious effort. Social signals are the basis of this highly effective communication. These signals are speech utterances, body movements such as gestures, manipulations of objects, and combinations thereof. For example, interlocutors typically position themselves in an F-formation (Goffman, 1963; Ciolek and Kendon, 1980; Kendon, 1990) and thereby signal to each other that they are part of that interaction. If another participant joins that interaction, the interlocutors integrate her in a new F-formation. The movements of each individual were comparably inconspicuous, but the intention for producing them was easily recognizable to the recipient. Humans use these signals intuitively and without conscious awareness. But in order to enable a robot to understand and respond appropriately to social signals, their form and function have to be made explicit. This research topic presents methods for identifying, understanding, and applying social signals in human–machine interaction.

Social signals are essentially multimodal but the analysis of human communication in human–machine interaction is often limited to the literal content of verbal utterances. For example, emotion has often been regarded as separate information that is specifically transferred through non-verbal signals, e.g., smiling. But Mehu argues that emotion is an inherent property of any social signal. The addressee would use the signal's emotional and literal content for determining how to respond to it. Identifying a signal's content requires combining and interpreting information from several modalities, taking into account the observer's prior experience. For example, Saegusa et al. show that a smiley next to a text message alters its perceived earnestness but its effect was more pronounced in hearing than non-hearing participants. Children also rely on multimodal signals for learning new words for objects. Hung et al. demonstrate that the children's strategic use of pointing gestures and spoken words depends on their linguistic experience, in particular if the gestures' reliability has been manipulated. Robotic recognizers have to combine data from sensors such as cameras and microphones for identifying objects and actions; a human is perceived as an entity with properties such as distance, body direction, and recent utterances. Similar to human observers, a robot requires detailed prior knowledge about social signals in order to interpret them. In a so-called “Ghost-in-the-Machine” study, Loth et al. show that human participants can identify social signals from the recognizer data of a robotic bartender. The study also shows that non-verbal signals were most important for initiating an interaction, whereas verbal signals were most important when placing an order. Multimodal signals unfold over time, and some features are available earlier than others. For example, the speaker's eye gaze reliably indicates the target of a selection task and preceded corresponding verbal utterances by almost 2 s in Huang et al.'s study of dyadic interactions. Thus, humans and robots can use this time for forming expectations about the verbal utterance and planning ahead. Understanding social signals is important for fulfilling a task, e.g., serving a drink. In addition to task performance, Shalev and Oron-Gilead argue that social signals are also crucial in regulating the assertiveness of companion robots, i.e., should the robot take the initiative or wait for being prompted.

Industrial robots do not socially interact with humans but operate as a tool repeating precisely the same actions. Sciutti et al. suggest to use the robot's ability to exactly reproduce behavior in order to investigate social signals in controlled natural settings, e.g. what kind of object manipulation a participant expects from a hand position. Furthermore, this enables research in dynamic environments, allowing Katevas et al. to investigate social signals with a robot stand-up comedian. They found that the robot's gaze behavior was an important signal for eliciting laughter. In contrast to comedy, questionnaires of the US census have been standardized with the aim of eliciting accurate responses independently of the interviewer's performance. However, Conrad et al. show that the verbal skills but not the facial animations of a virtual interviewer influence the accuracy of the participants' responses.

Interactions can and often do go wrong. However, if problems are repaired swiftly, the interaction is still perceived as smooth. Schegloff (Schegloff et al., 1977; Schegloff, 1992) argued that the speaker can repair a problem in her own utterance immediately (first position repair) or the hearer would try to initiate the repair (second position repair). In a third position repair, the hearer's response revealed a problem to the speaker allowing her to repair this in her next turn. Importantly, repairs require that the problem has been detected in the first place. After analyzing video recordings of human–robot interactions, Giuliani et al. conclude that users initially stopped moving when they encountered a problem. This could be used as a signal for the robot to initiate a first position repair immediately. The user's second position repairs involved many head gestures and lots of smiling signaling the robot that there was a problem. Some of the speakers' behaviors typically synchronize during an interaction such that a de-synchronization can indicate a communication error. For example, Andrist et al. show that the speakers' eye gazes typically settle on particular objects in a selection task. A deviation from this pattern indicates that a problem in the communication had occurred which required an explicit repair later in the interaction. Thus, detecting this cue allows both humans and robots to initiate a first position repair and resolve the problem instantly. Similar to gaze behavior, body movements synchronize during an interaction. Avril et al. augment a play session of children and their care-givers with sensors typically used in human–machine interaction. They show that prolonged periods of avoidance behaviors and asynchrony of body movements could indicate severe conditions such as child neglect.

All studies in this research topic underscored the fact that human communication is based on the exchange of social signals that are essentially multimodal. If these signals deviated from expected patterns, this indicated problems in the communication. The pattern of deviation identified the type of problem suggesting how to repair it. Furthermore, the absence of social signals can indicate severe psychological conditions. Thus, social signals are highly diagnostic, both in “normal” and problematic communication. They provide intuitive means for controlling the robot's current task and its relation to its user. However, understanding and producing social signals depends on prior knowledge in both humans and robots. To summarize, this research topic combines the research of psychologists and robot designers to contribute to our understanding of social signals and applies these insights to human–machine interaction.

## Author contributions

JDR and SL wrote, discussed, and revised several drafts before approving the final version.

## Funding

This research/work was supported by the Cluster of Excellence Cognitive Interaction Technology “CITEC” (EXC 277) at Bielefeld University, which is funded by the German Research Foundation (DFG).

### Conflict of interest statement

The authors declare that the research was conducted in the absence of any commercial or financial relationships that could be construed as a potential conflict of interest.
